# Sequential Dermoscopy and Reflectance Confocal Microscopy Paired With Pigmented Lesion Assay Gene Expression Profiling for In Vivo Monitoring of Multiple Halo Nevi on an Adult Patient

**DOI:** 10.7759/cureus.49465

**Published:** 2023-11-26

**Authors:** Joanna Ludzik, Emilie A Foltz, Jordan Gillespie, Alexander Witkowski

**Affiliations:** 1 Dermatology, Oregon Health & Science University, Portland, USA; 2 Dermatology, Washington State University Elson S. Floyd College of Medicine, Spokane, USA

**Keywords:** pigmented lesion assay, in-vivo, gene expression profiling, halo nevi, dermoscopy, confocal, reflectance confocal microscopy, halo nevus

## Abstract

The halo nevus is characterized by a ring of depigmentation appearing around an acquired or congenital melanocytic nevus. When observed in children, halo nevi are generally not a cause of concern. However, adult-onset halo nevi have an associated risk of primary cutaneous melanoma that corresponds to the risk of melanoma in patients with atypical nevi or a personal/familial history of melanoma. Thus, new-onset halo nevi in adults requires close follow-up and monitoring for malignancy. Herein we present a case of an adult patient who received sequential digital dermoscopy, reflectance confocal microscopy, and pigmented lesion assay gene expression profiling to monitor two halo nevi over a three-month period.

## Introduction

The halo nevus (HN), also called leukoderma acquisitum centrifugum, is characterized by a ring of depigmentation appearing around an acquired or congenital melanocytic nevus. HNs are most frequently found on the trunk and multiple lesions are present in approximately half of all cases. Clinically, the width of the halo is variable but of uniform radial distance from the center nevus that presents as a tan, pink, or brown uniformly colored round or oval macule [1]. In spite of the fact that most of the cases are idiopathic, sunburn and local skin trauma are suggested triggers that can lead to the initiation of self-reactivity. With time, the melanocytic lesion located in the center fades away and eventually disappears completely [2]. Vular et al. also reported that the halo phenomenon can rarely be seen around seborrheic keratoses, and may arise without an associated autoimmune disease or an underlying malignancy. The proposed pathophysiology is a result of an autoimmune response against melanocytes in which cytotoxic T-lymphocytes and antibodies to melanocytes (specifically IgM) cause depigmentation [3].

When observed in children, HNs are not a cause of concern, although De Schrijver et al. indicate that a biopsy is not needed if the pigmented part is regular in appearance; however, a biopsy might be justified in the presence of atypical features, as a halo phenomenon can be seen also in melanocytic lesion dysplasia or, rarely, in melanoma [4].

One recent multicenter retrospective study showed that adult-onset HN was associated with a 1% risk of primary cutaneous melanoma one year after HN diagnosis. This corresponds to the risk of melanoma in patients with atypical nevi or a personal/familial history of melanoma. New-onset HN in adults requires close follow-up and monitoring for malignancy [5].

Contrary to invasive histopathologic analysis, reflectance confocal microscopy (RCM) is an in vivo approach that permits cellular resolution observation of skin lesions from the epidermis down to 300 micrometers. Though several reports have described the diagnostic accuracy of RCM in common cutaneous neoplasms, there are few studies utilizing RCM to describe HN [6]. Available studies focused on various atypical features that include pagetoid cells, non-edged papilla, junctional thickening, nucleated cells in the dermal papillae, and plump bright cells [6,7]. Additionally, when utilizing digital dermoscopic images (DDI) to classify HN, the classic globular and/or homogeneous patterns typically seen in many benign melanocytic nevi are observed. Though, sequential follow-up using DDI can help in the identification of changes in a nevus of interest. One study suggested that follow-up of HN using DDI may not correctly rule out melanoma since halo-like depigmentation can be found in both [8].

Another non-invasive tool that assists in the diagnosis of potentially malignant lesions is the 3-GEP pigmented lesion assay (3-GEP PLA). This test detects the presence of three genes associated with melanoma (LINC00518, PRAME, and TERT) using adhesive patch testing and has the potential to reduce unnecessary biopsies. 3-GEP PLAs intended to provide information on gene expression risk factors for melanoma in pigmented skin lesions. This approach has been validated against histopathology and has decreased the need for surgical biopsies of suspicious lesions [9]. As such, 3-GEP PLA represents another form of in vivo testing that can further categorize the nature of HN in abnormal settings. Herein we present a case of an adult patient who received sequential DDI and RCM to monitor two HNs over a three-month period.

## Case presentation

Baseline visit

**Figure 1:**
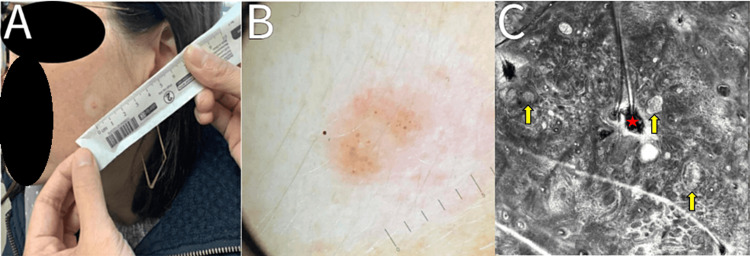
Halo nevus visualized on the left cheek at baseline visit. Panel A indicates the location of a 5 mm pink-brown papule with a hypopigmented rim alongside a metric ruler. Panel B shows a dermoscopy of the lesion, with a globular-homogeneous pattern network and an absence of vessels. Panel C highlights reflectance confocal microscopy-enabled visualization of a clod pattern representing homogeneous dermal nests of melanocytes (yellow arrows) and a hair follicle (red star) without atypical cells.

**Figure 2:**
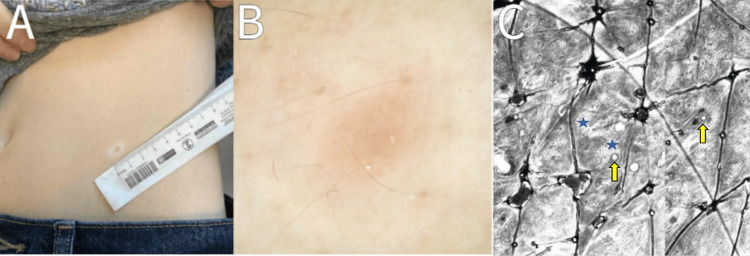
Halo nevus visualized on the left lower abdomen at baseline visit. Panel A indicates the location of the 7 mm pink-brown papule with a surrounding hypopigmented ring 1 cm in diameter. Panel B demonstrates an absence of vessels and a globular-homogeneous pattern network on dermoscopy. Panel C is a reflectance confocal microscopy-captured image of a honeycombed epidermis with milia-like cysts (yellow arrows) and areas of increased collagen (blue stars). There is an absence of pagetoid cells.

A 40-year-old Fitzpatrick skin type I female presented to the dermatology clinic for a full-body skin examination, requesting that extra attention be paid to a spot on her face. The patient’s primary concern was a slightly elevated papule on her left cheek that demonstrated observable self-reported changes within the last year. She reported that the pink-to-brown papule was present for the entirety of her life, yet it recently developed a surrounding white circle that had been increasing in size. Of note, she stated that a pigmented papule on her left abdomen had a similar surrounding white ring that had just begun. She denied itching of the lesions and denied growth of the pigmented portions of both lesions (Figures 1, 2).

On clinical examination, a 5 mm slightly elevated pink-to-brown-colored papule with a hypopigmented rim was present on the left cheek. Dermoscopically, the lesion exhibited a globular homogeneous pattern with an absence of vessels (Table 1, Figure 1). RCM demonstrated features similar to that visualized on the lesion on the abdomen, with a regular, predominantly honeycombed epidermis, a meshwork and clod pattern present at the dermal-epidermal junction, and an absence of dendritic and pagetoid cells. Heterogenous nests were present as well as sparse inflammatory cells (Table 2). These findings further supported the diagnosis of a compound melanocytic nevus with an encompassing halo rim and concern for melanoma was low.

**Table 1:** Pattern of pigmented network observed within lesions with the application of dermoscopy.

Pattern	Left abdomen		Left cheek	
	Baseline	Month 3	Baseline	Month 3
Globular	Absent	Absent	Absent	Absent
Reticular	Absent	Absent	Absent	Absent
Homogeneous	Absent	Absent	Absent	Absent
Globular-homogeneous	Present	Present	Present	Present
Reticular-homogeneous	Absent	Absent	Absent	Absent

**Table 2:** Epidermal, junctional, and dermal visualization of cellular features enabled with reflectance confocal microscopy.

Feature		Left abdomen		Left cheek	
		Baseline	Month 3	Baseline	Month 3
Superficial epidermal layers					
	Honeycombed	Present	Present	Present	Present
	Honeycombed atypical	Absent	Absent	Absent	Absent
	Honeycombed broadened	Present	Present	Absent	Absent
	Cobblestone	Absent	Absent	Absent	Absent
	Cobblestone atypical	Absent	Absent	Absent	Absent
	Cobblestone with small nucleated cells	Absent	Absent	Absent	Absent
	Roundish pagetoid cells	Absent	Absent	Absent	Absent
	Dendritic pagetoid cells	Absent	Absent	Present	Present
Dermoepidermal junction					
	Edged papillae	Present	Present	Present	Present
	Nonedged papillae	Absent	Absent	Present	Present
	Junctional nests	Present	Present	Present	Present
	Junctional thickenings	Absent	Absent	Absent	Absent
	Sheet-like structures	Absent	Absent	Absent	Absent
Papillary dermis					
	Dense clusters	Absent	Absent	Absent	Absent
	Dishomogeneous nests	Absent	Absent	Present	Present
	Sparse cell nests	Present	Present	Present	Present
	Cerebriform nests	Absent	Absent	Absent	Absent
	Isolated cells in the papilla	Absent	Absent	Absent	Absent
	Plump bright cells	Present	Present	Present	Present
	Collagen reticulated	Present	Present	Present	Present
	Collagen bundles	Present	Present	Present	Present

The lesion on the left abdomen appeared as a 7 mm pink-brown papule with the presence of a 1 cm hypopigmented ring. Dermoscopy demonstrated an absence of vessels, thereby favoring the diagnosis of a nevus with a halo rim (Figure 2). As previously mentioned, adult-onset HN’s association with malignant melanoma indicates close monitoring of the lesion. To offer a non-invasive alternative to biopsy in this patient with one spot of concern, and a second identified by the physician, further visualization was conducted with RCM (Vivascope 1500; Caliber I.D., Rochester, NY). Cellular and sub-cellular imaging of the skin lesion was obtained with RCM, with four individual mosaics taken at the levels of the epidermis, dermal-epidermal junction, and upper dermis. The epidermal architecture was a regular, predominantly honeycombed pattern. Pagetoid cells were absent. The dermal-epidermal junction architecture was regular and composed of a ringed pattern. Atypical cells were absent. Inflammatory cells were present (Table 2). The features suggested that the skin lesion was a melanocytic nevus with a halo rim and concern for melanoma was low. Physical biopsy was not performed due to the lack of concerns for malignancy and close follow-up was recommended.

Clinical recommendations and consideration of the possible malignant potential of halo nevi governed the follow-up for this patient [4]. The patient was thereby recommended close monitoring of both lesions, including repeat imaging with RCM in three months.

Three-month follow-up visit

**Figure 3:**
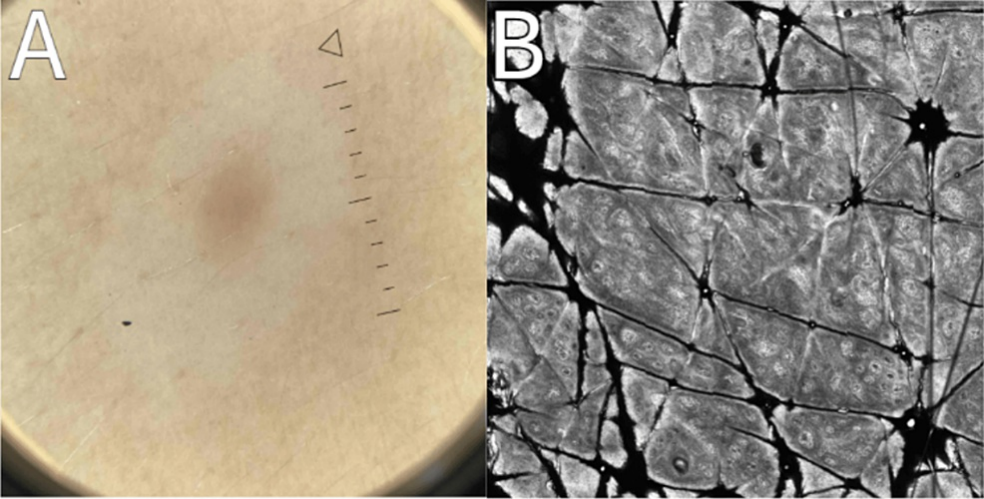
Follow-up imaging, three months from baseline, of the left lower abdomen halo nevus. Panel A demonstrates unchanged dermoscopic features of a globular-homogeneous pigment network and panel B showcases a regular epidermis with reflectance confocal microscopy, composed of a honeycomb pattern, and an absence of pagetoid cells.

**Figure 4:**
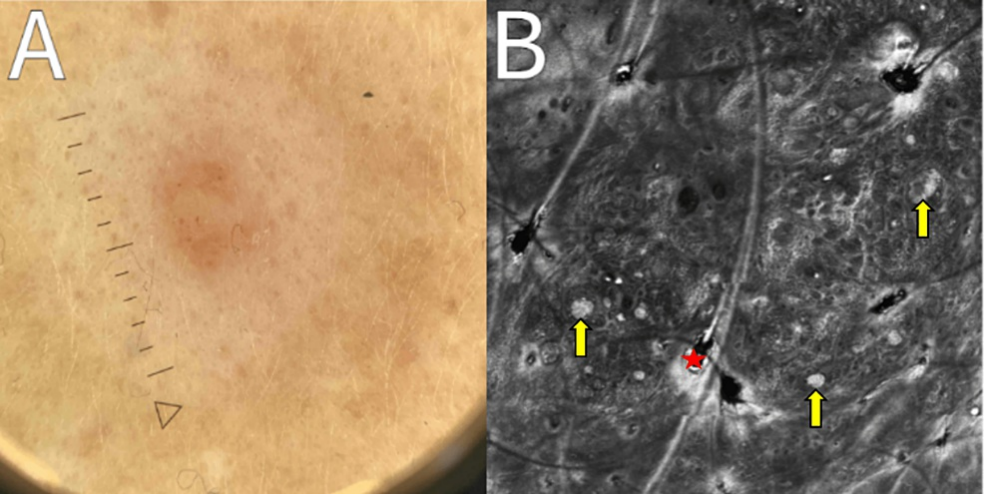
Follow-up imaging, three months from baseline, of the left cheek halo nevus. Panel A indicates unchanged dermoscopic features of a globular homogeneous network and an absence of vessels. Reflectance confocal microscopy imaging of the lesion is shown in panel B, consistent with previous findings of dermal nests (yellow arrows), a hair follicle (red star), and an absence of atypical cells.

Upon subsequent three-month follow-up, the patient reported no visible changes to the lesions. Clinical examination affirmed the patient’s reported findings in lesion size and pigment, and stable dermoscopy again suggested two melanocytic nevi with the presence of a globular-homogeneous network (Table 1; Figures 3, 4).

RCM images of both lesions remained stable, with a mild increase in dendritic pagetoid cells without nuclei in the epidermis, thickening at the dermal-epidermal junction, and inhomogeneous nests in the papillary dermis observed in both lesions (Table 2; Figure 4).

Due to the observed increased number of dendritic cells enabled by RCM, a non-invasive 3-GEP PLA was utilized to assess the malignant potential of both lesions. The results for both lesions were negative and we decided to continue with follow-up of the lesions every six months.

## Discussion

There are four clinical stages of HN beginning with a melanocytic nevus surrounded by a halo of depigmentation (Stage 1). Then a pink nevus surrounded by a halo of depigmentation develops (Stage 2) followed by a circular area of depigmentation with total/partial disappearance of the nevus (Stage 3). Finally, the skin appears normal after re-pigmentation of the halo (Stage 4) [4].

The duration of HN stage progression is widely variable, but usually no longer than a few months. For those HNs that progress to complete resolution (Stage IV), the average time is almost eight years. Education about the prolonged natural history of HN plays an important role in the clinical setting [10].

Given the higher risk of melanoma occurring in adults with HN [5], close monitoring is recommended. Previous studies have described the reliability of RCM in the evaluation of HN in consideration of the ground truth of histopathology and for this reason, the diagnosis was made based on clinical-dermoscopic confocal presentation [3]. Sequential imaging with dermoscopy and RCM in this patient’s case enabled visualization of features suggestive of two benign melanocytic nevi undergoing gradual regression and enabled our team to confidently provide safe and effective patient management [11]. In the case of 3-GEP PLA, expression of LINC00518, PRAME, and TERT promoter was not found, providing further reassurance of the current benign nature of both lesions and allowing for continued monitoring without an invasive biopsy within six months. In addition, a recent paper by Ruby et al. showed that negative PRAME immunostaining may be a reassuring finding to help differentiate HN from melanoma, which would have been considered in the setting of an invasive biopsy which was avoided due to lack of concerning RCM features [12].

Limitations of our study include a small sample size and the number of follow-up visits for this patient. Future studies applying these combined techniques for monitoring HN should consider a larger cohort of participants and with documented data from multiple follow-up visits.

## Conclusions

In this case, we present the first novel report of multiple HNs monitored sequentially with dermoscopy and RCM and augmented with 3-GEP PLA in one adult patient. This patient with multiple HNs at once was resistant to biopsy due to fear of scarring in cosmetically sensitive areas. This report proposes a multifactorial approach to sequential monitoring of these lesions with non-invasive modalities at both the cellular and genetic expression profile levels.

Combining these three in vivo approaches, we ultimately found that this patient’s HN was stable at the clinical-dermoscopic confocal and 3-GEP levels and deemed appropriate for non-invasive monitoring, thereby minimizing the biopsy burden both for the healthcare system and the patient.

We hereby propose a non-invasive alternative to histopathology for monitoring patients with multiple HNs using DDI, RCM, and 3-GEP PLA in order to reduce unnecessary invasive biopsies, especially in cosmetically sensitive areas.
